# Intraosseous mucoepidermoid carcinoma of the mandible

**DOI:** 10.3332/ecancer.2023.1599

**Published:** 2023-09-04

**Authors:** Lucas Avondet, Roque Adan, Barbara M Berenstein, Gonzalo Zeballos, Nicole Brasquet, Ángeles Da Silva, Candela Bazan, Gisela Coliva, Ximena Garcia

**Affiliations:** 1 Alexander Fleming Cancer Institute, Head and Neck Unit, 1180 Cramer, Buenos Aires 3201, Argentina; 2 Alexander Fleming Cancer Institute, Pathological Anatomy Service, 1180 Cramer, Buenos Aires 3201, Argentina

**Keywords:** salivary glands, mucoepidermoid carcinoma, intraosseous lesions, mandibular neoplasms

## Abstract

**Background:**

Mucoepidermoid carcinoma starts in the salivary glands and accounts for 5%–10% of all salivary gland tumours. Its intraosseous occurrence is rare and only accounts for 2%–3% of all mucoepidermoid carcinomas. This neoplasm often follows a long and indolent course. Also, given its ambiguous presentation and similarities to other dental pathologies, it often has a late diagnosis. In this instance, we present the case of a patient with an asymptomatic mandibular lesion, who underwent mandibular resection and reconstruction with fibula free flap.

**Case report:**

A 32-year-old male patient reported discomfort when chewing, which was attributable to a self-detected mass localised in proximity to teeth 47 and 48. The lesion presented as a slight swelling without clear expression in the oral cavity mucosa. The rest of the physical examination revealed no abnormalities. Both the panoramic radiograph and computed tomography of the maxillary bones revealed lytic lesions in proximity to teeth 47 and 48, close to the mandibular angle. An incisional biopsy was performed, for which the pathological anatomy showed low-grade mucoepidermoid carcinoma. A resection was then performed, which involved a right hemimandibulectomy with ipsilateral cervical lymphadenectomy. The reconstruction was performed with a right fibula-free flap. Upon histological evaluation of the surgical specimen, an intermediate-grade mucoepidermoid carcinoma was found. The patient presented good post-operative evolution. Following a multidisciplinary assessment, the use of adjuvant radiation therapy was deemed necessary. The patient currently presents good evolution and has regular check-ups.

**Conclusion:**

Intraosseous mucoepidermoid carcinoma is a rare salivary gland tumour. Given its low frequency, there are no studies that accurately describe its biological behaviour and prognosis.

## Introduction

Mucoepidermoid carcinoma is the most common salivary gland malignant neoplasm in adults. It was first described by Stewart *et al* [1] as a neoplasm with epidermal and mucus-secreting elements [2]. This pathology accounts for around 5%–10% of all salivary gland neoplasms [3] and most often affects the major salivary glands, and the parotid gland in particular. When it affects the minor salivary glands, it occurs most often in the hard palate, retromolar trigone, oral mucosa, tongue, lips, floor of the mouth and larynx [4, 5].

Intraosseous mucoepidermoid carcinoma is rare and only accounts for 2%–3% of all reported cases of mucoepidermoid carcinoma [2–6]. It shows a slight predilection for women and commonly affects the mandible more than the maxillary bone, with the premolar angle region being the most common site. Cases have been reported in all age groups (from 1 to 78 years). However, the majority are detected in those aged 30–50 [2, 3]. This tumour is rare in the paediatric age group, where the mandibular-maxillary bone case ratio is 1:1.

The origin of these tumours is controversial. It has been suggested that this neoplasm may originate from ectopic salivary gland tissue, submucosal glands with intraosseous extension or the transformation of odontogenic mucous cells [2–6]. It most commonly presents as a gradually increasing painless mass and is detected during self-examination or a finding during dental procedures [7]. In some cases, it can manifest as pain, trismus, paraesthesia and mobility or loss of teeth.

On imaging, it usually appears as a well-circumscribed unilocular or multi-locular lesion with well-defined borders. As its clinical and radiographic expression does not differentiate it from other benign odontogenic lesions, a biopsy or cytology must be performed for its accurate diagnosis [6].

Radical resection with adequate oncologic margins is the treatment modality that provides the best results [2, 3, 6]. However, there are no treatment guidelines for this neoplasm.

## Case report

**Figure 1.**
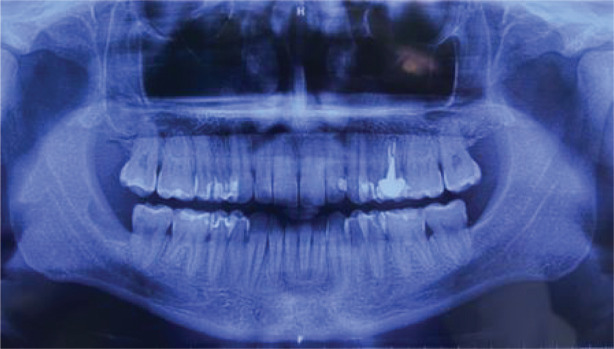
Panoramic radiograph of the maxillary. A radiolucent cystic and osteolytic lesion localised in the retromolar trigone can be seen in proximity to teeth 47 and 48.

**Figure 2.**
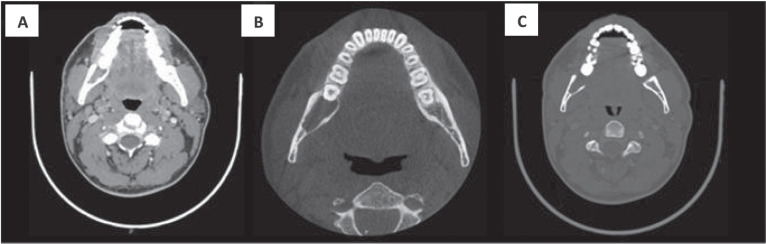
CT with intravenous contrast. Both the soft tissue window (a) and bone windows (b–c), shows an osteolytic lesion with right mandibular body and ramus involvement, localised in the retromolar space behind tooth number 48. The lesion features soft tissue components and presents lingual cortical disruption. It shows the involvement of the adjacent inferior alveolar nerve canal. It measures approximately 29 × 23 × 11 mm in size.

A 32-year-old male patient reported discomfort when chewing, which was attributable to a self-detected mass localised in proximity to teeth 47 and 48. The lesion presented as a slight swelling of the oral cavity mucosa in the right retromolar space. However, there was no mucosal epithelium disruption or involvement. Various lymph node levels were assessed, and no adenopathy was found. The rest of the physical examination revealed no abnormalities. The panoramic radiograph of the maxillary bones showed a radiolucent lytic area in proximity to teeth 47 and 48 (Figure 1). The computed tomography (CT) (Figure 2) confirmed the presence of the 29 × 23 × 11 mm lesion described on the radiograph, which showed lingual cortical disruption and mandibular body and ramus involvement. The incisional biopsy found a lesion consistent with low-grade mucoepidermoid carcinoma.

**Figure 3.**
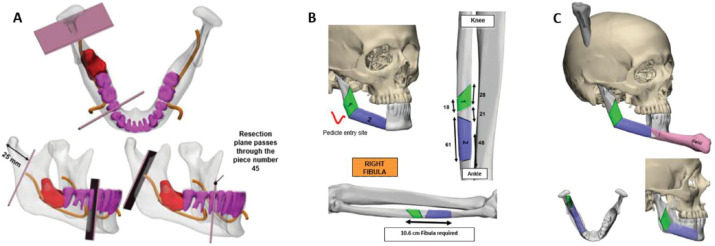
3D Stereo-lithographic planning. (a) Lesion in red in relation to the inferior alveolar nerve and teeth. Cutting guides with adequate oncological margins and prefabricated angulation for their subsequent reconstruction were provided. (b and c) Cutting lines made on the right fibula for the incorporation of bone fragments, an accurate subsequent osteosynthesis and reconstruction of the new mandible.

Based on this information and following the multidisciplinary assessment, we planned the partial mandibular resection with oncological margins and the reconstruction using a microvascular osteomyocutaneous fibula flap to reconstruct the shape and height of the mandible. The flap not only made it possible to reconstruct the maxillofacial silhouette, but to also provide the possibility of future dental implants and rehabilitation. The resection range and the reconstruction of the length and angle of the fibula were prefabricated using 3D stereo-lithographic reconstruction models (Figure 3).

**Figure 4.**
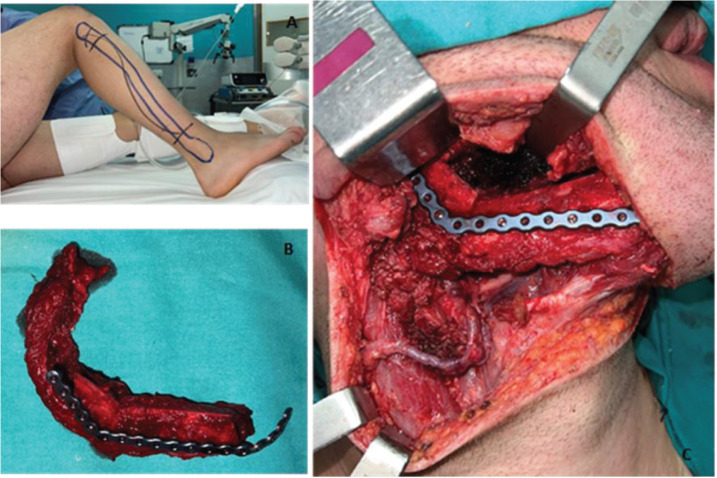
(a) Layout established for the free fibula flap approach. (b) Resected fibular fragment alongside the peroneal muscle and vascular bundle. The bone fragments were moulded and fixed to the titanium reconstruction plate. (c) Highlights the anastomosis of the peroneal veins to the facial artery, the external jugular vein, and the facial vein. It also shows its fixation to the remaining right mandible using a titanium reconstruction plate.

The patient underwent radical tumour resection guided by digital design. A standard trans-cervical approach was used. The ipsilateral submandibular gland and the ipsilateral cervical lymph nodes (Levels I–III) were removed. A free fibula flap was used to reconstruct the mandible with a reconstruction plate and 2.0 screws. The peroneal pedicle was anastomosed to the facial artery, the external jugular vein, and the facial vein. The mucosa and skin closure were performed without any complications. (Figure 4)

**Figure 5.**
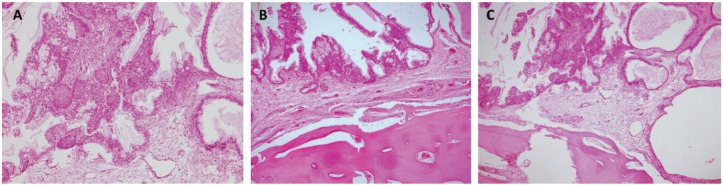
Histopathology. Haematoxylin and eosin stain (10×). (a) Atypical epithelial proliferation consisting of mucinous, intermediate and squamous cells. The cells described form solid nests that line glandular spaces, some with cystic dilations and extracellular mucin. (b and c) Epithelial proliferation with similar characteristics and active compact bone tissue involvement.

**Figure 6.**
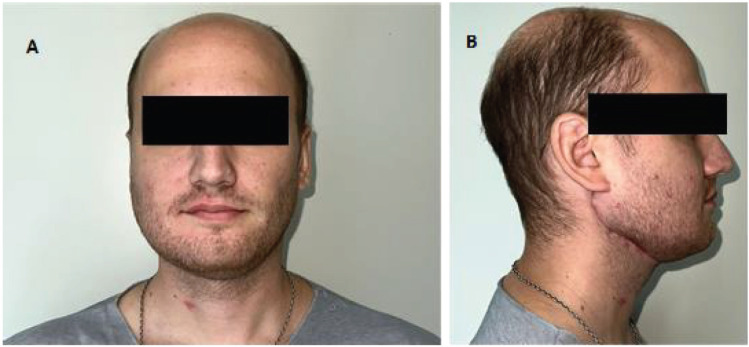
Second post-operative month. Both figures (a) and (b) show the correct lateral and anterior projection of the reconstructed mandible. The patient has on-going masticatory function rehabilitation exercises.

The routine postoperative pathologic assessment found the specimen had a cystic structure lined with myxoid, tall columnar and squamous epithelial cells. The intermediate-grade mucoepidermoid carcinoma diagnosis was confirmed based on histopathology (Figure 5). The histopathological characteristics only showed reactive lymphoid hyperplasia; no metastatic lymph node involvement was found on assessment. Following the multidisciplinary assessment, the use of postoperative adjuvant radiation therapy was deemed necessary given the histological grading of the lesion. The patient currently has regular clinical check-ups (every 2 months) without evidence of recurrence (Disease-free period: 6 months). The patient does not have any major therapeutic procedure side effects and has on-going rehabilitation (Figure 6).

## Discussion

Intraosseous mucoepidermoid carcinoma is a rare neoplasm that only accounts for 2%–3% of all mucoepidermoid carcinomas [3]. It is most commonly localised in the posterior region of the mandible and usually affects more women. Although there are case reports for various age groups, it most often presents in the third and fifth decade of life [2, 3].

Its aetiology and pathogenesis are unknown. Various theories have been published to try and explain its origin [3, 8, 10, 11]. One of the potential origins is the neoplastic transformation of mucous secretory cells found in the pluripotential epithelial lining of dentigerous cysts associated with impacted third molars. A second proposed theory is the entrapment of retromolar mucous glands during mandible development, thus forming mucous secretory cell nests that may undergo neoplastic alteration. A third hypothesis states that it originates in ectopic glandular tissue within the mandibular canal during development. Bouquot *et al* [9] have reported the presence of intraosseous salivary gland tissue in 0.3% of the maxillary bones studied, thus providing further evidence of the possible origin of these tumours.

Traditionally, mucoepidermoid carcinomas are histologically classified into three grades based on the number of cystic cells, cellular atypia grading and the number of mucosal, epidermoid and intermediate cells. Low-grade tumours present as multiple well-differentiated cystic formations, with minimal cellular atypia and a relatively high proportion of mucosal cells. High-grade tumours consist of solid islands of squamous or intermediate cells and have considerable pleomorphism and mitotic activity. The production of mucous secretions is relatively infrequent, and it is thereby often difficult to differentiate it from a squamous cell tumour. Intermediate-grade tumours have characteristics that rank them between low-and high-grade tumours [12, 13].

Intraosseous mucoepidermoid carcinomas are most common in young adults and show a slight predilection for women. They are three times more common in the mandible than the maxillary bones and often affect the molars and mandibular rami area. They usually present as a mass or formation causing cortical swelling, which is often found during a radiographic examination or dental procedure [13]. Pain, trismus and paraesthesia have also been reported and often relate to the size and location of the lesion. The presence of metastasis has also been reported in approximately 12% of all intraosseous mucoepidermoid carcinomas, particularly in the regional lymph nodes [13].

Further imaging studies play a vital role in diagnosing this pathology. However, given its non-specific features, like the presence of peripheral sclerotic tissue, radiolucency, mixed internal structures and unilocular or multi-locular patterns, it is difficult to differentiate it from other lesions like ameloblastoma, odontogenic cysts and keratocystic odontogenic tumours [13, 14]. Despite these difficulties in its accurate assessment, panoramic radiograph and CT studies are essential.

Fine needle aspiration is a highly used and recommended diagnostic methodology in this salivary gland pathology. With its high sensitivity and specificity, it can also be used for the cytological diagnosis of these neoplasms [3].

The following criteria are accepted for the diagnosis of intraosseous mucoepidermoid carcinoma: a) the lesion must have epithelial and mucinous components; b) it may cause bone margin destruction and/or cortical bone expansion; c) the cortical plates are intact; d) an absence of accompanying lesion in the salivary glands; e) the exclusion of any other primary tumours; and f) histopathological findings that confirm the tumour [15]. The staging system for intraosseous mucoepidermoid carcinomas depends on the condition of the overlying bone. In Stage I, the lesion has an intact cortical border and no evidence of bone expansion; in Stage II, the lesions are surrounded by intact bone showing some expansion; and in Stage III, the lesions are associated with cortical perforation, breakdown of the overlying periosteum or nodal spread [2, 3, 10].

Surgery remains the mainstay of intraosseous mucoepidermoid carcinoma treatment. In a review of 64 patients, Brookstone and Huvos [10] found recurrence rates of 40% following conservative surgical modalities, like enucleation, curettage, marsupialisation and marginal resection with or without adjuvant therapy. However, in the patient group that underwent radical resection, with or without associated adjuvant therapy, only 4% experienced recurrence [11–13]. There are no treatment guidelines that provide lymphadenectomy guidance with a significant level of evidence in cases where the neck is clinically negative. For the foregoing reasons, it is recommended that a lymphadenectomy be performed on the primary lesion level in high-grade intraosseous mucoepidermoid carcinomas. In case of regional lymph node involvement, a lymphadenectomy will be performed on the corresponding levels.

There is also no accumulated evidence on adjuvant guidelines. However, radiation therapy with/without chemotherapy is recommended in cases of high-grade mucoepidermoid carcinomas [2, 3, 10, 13].

Because of the various treatment approaches and biological features, the prognosis of intraosseous mucoepidermoid carcinoma is difficult to predict. Various factors are associated with its prognosis, including gender, histological grade, surgical approach and the regional ganglionic condition. Let’s remember, this neoplasm has the potential to recur and metastasise. It is therefore advisable to establish long-term follow-ups [16].

## Conclusion

Intraosseous mucoepidermoid carcinoma is a rare malignant head and neck tumour. It tends to be a low-grade histological lesion with a low level of aggressiveness. However, there are reports of local aggressiveness, recurrence and metastasis, especially on a regional level. Its diagnosis is challenging due to the difficulty in differentiating it from other lesions. Nevertheless, the final diagnosis is usually made using histology and additional imaging studies. Resection with adequate oncologic margins is the mainstay of treatment. Owing to its potential recurrence, even long after the operation itself, a long-term follow-up plan must be implemented. Also, due to its rarity and controversial issues, studies must be conducted to fully elucidate the appropriate management of this pathology and establish evidence-based treatment guidelines.

## Conflicts of interest

There are no conflicts of interest to declare in this publication.

## Funding

The listed authors received no funding to prepare this paper.

## Informed consent

The patient's prior informed consent was obtained for this Case Report.
